# Molecular Dynamics Investigation of CSH/SiO_2_ Interface Degradation in High-Temperature and Water-Rich Environments

**DOI:** 10.3390/ma19112295

**Published:** 2026-05-28

**Authors:** Lianzhen Zhang, Yiwei Hu, Qingsong Zhang, Changxin Huang, Liangchao Zou, Zhipeng Li, Runan Wang, Congjian Feng, Mingchen Li, Zongjian Yang

**Affiliations:** 1 College of Pipeline and Civil Engineering, China University of Petroleum, 66# Changjiang West Road, Qingdao 266580, China; 2 State Key Laboratory for Tunnel Engineering, School of Civil Engineering, Shandong University, 17923# Jingshi Road, Jinan 250012, China; 3 School of Architecture and the Built Environment, KTH Royal Institute of Technology, 100 44 Stockholm, Sweden; 4 School of Transportation and Civil Engineering, Shandong Jiaotong University, 5001# Haitang Road, Jinan 250357, China

**Keywords:** molecular dynamics simulations, CSH/SiO_2_ interface, high-temperature environment, water-rich environment, interface degradation mechanism

## Abstract

**Highlights:**

- MD simulations reveal CSH/SiO_2_ interface degradation under heat–water coupling.
- Thermal excitation enhances atomic mobility, disrupting Ca-O bond and H-bond networks.
- High temperature induces ITZ expansion and structural loosening of the interface.
- Water competes for active sites, turning direct CSH/SiO_2_ bonding into water-mediated.
- High temperature acts as a catalyst for deeper water molecule penetration into CSH.

**Abstract:**

As the critical weak link in grouting reinforcement systems, the interfacial adhesion between cementitious grout and rock minerals is highly susceptible to performance degradation under high-temperature and water-rich conditions. In this paper, molecular dynamics simulations were performed across a temperature range of 293 K to 368 K to systematically investigate the effects of high-temperature and water-rich environments on the mechanical response, bonding structure, and dynamic behavior of the grout–rock interface. All simulations were performed using the LAMMPS package with the ClayFF force field. Two interface models, including a CSH/SiO_2_ direct-contact model and a CSH/H_2_O/SiO_2_ water-containing model, were constructed and subjected to uniaxial tensile tests. Key findings are as follows: (i) The tensile strength and interaction energy of the CSH/SiO_2_ interface exhibit distinct thermal degradation characteristics. The tensile strength decreases by 32.57%, and the interaction energy by 15.78% when the temperature rises from 293 K to 368 K. High temperatures induce expansion of the interface transition zone from 2.74 Å to 4.60 Å and loosening of the interface structure. (ii) High temperatures intensify atomic diffusion at the interface. The number and stability of Ca-O bonds and hydrogen bonds formed between CSH and SiO_2_ are reduced, leading to a decline in interfacial adhesion. (iii) The presence of an interfacial water layer significantly impairs the tensile strength and interaction energy of the interface. Compared with the direct-contact interface, the interaction energy is reduced by 38% at 293 K, and the tensile strength decreases by 73.58%. Water molecules in the solution compete for bonding sites of hydrogen bonds and Ca-O bonds at the interface, weakening the direct interaction between CSH and SiO_2_ and transforming it into an indirect interaction mediated by water molecules.

## 1. Introduction

As underground space development advances toward greater depths, underground projects face increasingly severe challenges posed by high geothermal temperatures and water-rich fractured rock mass environments [1,2]. High-temperature fissure water inrush disasters occur frequently and become one of the major disasters in deep-buried underground construction projects [3,4]. Grouting reinforcement is a critical technology for ensuring the integrity and impermeability of fractured rock masses [5,6]. Cement-based materials, renowned for their high strength, durability, and cost-effectiveness, have become the material of choice for grouting [7,8]. The grout–rock interface, as the weakest link in the grouting reinforcement system, directly governs the efficacy of grouting and overall structural safety [9,10,11]. However, the synergistic effects of high temperature and water-rich conditions induce the degradation of the intrinsic properties of cement-based materials, resulting in a significant loss of interfacial integrity at the grout–rock boundary, severely compromising the service life of underground engineering structures [12,13,14].

To date, numerous scholars have obtained extensive findings on the mechanical properties of the grout–rock bonding interface through macroscopic experiments [15,16,17,18]. Koupouli et al. [15] noted that the grout–rock interface exhibits extremely weak adhesion in the early stages, functioning as a vulnerable zone within the composite system. Regarding the influence of environmental factors, multiple studies have confirmed the degradation effects of high temperature and the presence of water on interfacial bonding strength. Fang et al. [16] found that while high curing temperatures can accelerate the hydration rate of cement, the shear strength is lower than that of specimens cured at ambient temperature, exhibiting a temperature inversion phenomenon in the strength of cementitious materials. Hochstein et al. [17] further corroborated this, noting that high geothermal environments reduce the bonding strength between concrete and rock. Through temperature–humidity coupling experiments, Tang et al. [18] found that high temperature and humidity induce the microstructural degradation in concrete, leading to a significant decrease in interfacial bonding strength. Collectively, these studies have clarified the detrimental impacts of high-temperature and water-rich environments on the macroscopic mechanical properties and interfacial adhesion behavior of the grout–rock interface.

These experimental studies have primarily focused on the influence of external environments on the macroscopic mechanical properties of the grout–rock interface, while lacking an in-depth understanding of the intrinsic mechanisms governing interfacial bond evolution and structural degradation. To reveal the mechanisms of interaction at the grout–rock interface under varying temperature conditions, it is typically necessary to investigate its nanoscale interaction modes. However, current macroscopic experimental approaches are largely limited in their ability to yield insights at the molecular level [19,20,21]. The emergence of molecular dynamics (MD) simulations has provided a robust tool for dissecting the fundamental nature of interfaces at the atomic scale [22,23].

Calcium silicate hydrate (CSH), which accounts for approximately 60% of the volume of cement hydration products, is commonly employed as a representative model for cement grout [24,25,26]. Quartz, as the dominant mineral component of rocks such as granite and sandstone in grouting engineering projects [27,28,29], was selected in this study as the representative mineral model for the rock surface, with the SiO_2_ model adopted to simulate its crystalline structure. Existing research on CSH has centered on its structural characteristics [30,31], mechanical properties [32,33], and interfacial behaviors [34,35,36]. Yang et al. [31] revealed the interfacial stabilization mechanisms between CSH and minerals such as silica and albite at the atomic scale. The results confirmed that Ca^2+^ bridging, hydrogen bond networks, and chemical bonding constitute the core of interfacial adhesion. This conclusion was further validated by Ming-Feng et al. [34] through tensile tests on the CSH/SiO_2_ interface. Zhang et al. [35] investigated the interfacial behavior of CSH/polymer/SiO_2_ composite structures within the temperature range of 300–400 K, noting that high temperatures weaken interfacial bonding interactions and reduce adhesion strength. Jiang et al. [36] examined the interfacial behavior of CSH/NASH under different temperature conditions and in the presence of an interfacial water layer. Their results demonstrated that the presence of interfacial water modulates the dynamics of interfacial interactions while concurrently diminishing both the bonding strength and ductility of the interface. Furthermore, in actual deep underground environments, the complexity of groundwater introduces chemically aggressive ions, which can further degrade the mechanical properties of cementitious interfaces. From a micro-perspective, sulfate invasion triggers chemical reactions with hydration products, forming expansive phases (e.g., ettringite) that induce micro-cracking and interfacial decohesion [37]. Meanwhile, chloride ions compete for adsorption sites and disrupt the local electrostatic and hydrogen-bonding networks at the interface, reducing interfacial bonding strength [38]. As a first step to decouple these complex effects, this study intentionally excludes chemical ions to isolate the coupled physical degradation of the pristine interface induced by high temperature and water-rich conditions. This provides a critical baseline for subsequent studies investigating the synergistic effects of chemical attacks and physical factors.

These studies have laid a theoretical foundation for investigating the performance of the grout–rock (CSH/SiO_2_) interface. However, existing microscopic research predominantly focuses on polymer-containing composite interface systems and primarily explores interface evolution under the action of a single environmental factor. In the actual grouting environment of deep tunnels, the grout–rock interface is consistently subjected to the dual coupling effect of high-temperature and water-rich conditions. There remains a lack of targeted research on the atomic-scale degradation mechanism of the cement-based grout–rock interface under high-temperature and water-rich environments.

Herein, molecular dynamics simulations were employed to investigate the effects of different temperature environments and interfacial water layer intervention on the mechanical behavior and bonding performance of the grout–rock interface in grouting engineering. Two models were constructed: a CSH/SiO_2_ direct contact model to simulate the direct grout–rock contact region in grouting interfaces, and a CSH/H_2_O/SiO_2_ water-containing interface model to represent the grout–water–rock zone. Uniaxial tensile simulations were conducted to explore the interfacial mechanical responses of the two composite models across a temperature range of 293 K to 368 K. By comparing the interfacial behavior of the models under different temperatures and interfacial water layer states, the interfacial interactions, local bonding characteristics, and atomic dynamic behaviors were analyzed in depth. The fundamental mechanisms governing the degradation of the grout–rock interface are elucidated at the atomic scale, providing a theoretical framework for the microstructural optimization of cementitious grouting materials in deep high-temperature tunnels.

## 2. Model Construction and Simulation Methods

### 2.1. Model Construction

**Figure 1:**
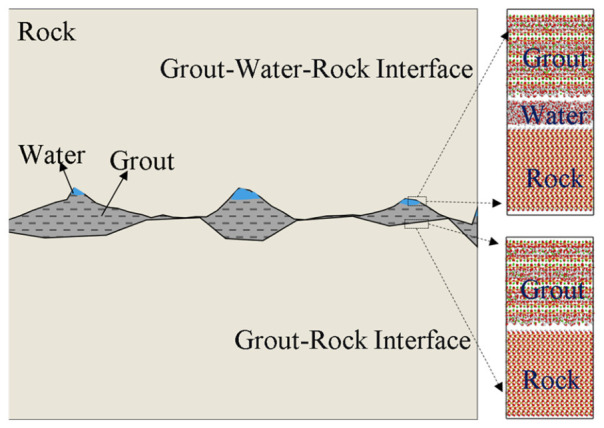
Distribution of grout within water-rich fractured rock masses.

In the grouting of water-rich fractured rock masses, the grout often fails to achieve 100% fracture filling due to the influence of fracture geometry. Consequently, the grout may either be in direct contact with the rock mass on both sides of the fracture, or a water-filled gap may exist between the grout and the rock. The degree of fracture filling directly dictates the effectiveness of the grouting reinforcement. In this study, a CSH/SiO_2_ direct-contact model was constructed to simulate the condition where the grout completely fills the fracture, while a CSH/H_2_O/SiO_2_ water-containing interface model was developed to represent the incomplete filling state. The macroscopic grouting states in water-rich fractured rock masses and the corresponding molecular models for the two types of interfaces are depicted in Figure 1.

In this study, all atomistic model constructions were executed using Visual Molecular Dynamics (VMD) [39]. All molecular dynamics simulations were performed using the Large-scale Atomic/Molecular Massively Parallel Simulator 8 February 2023 (LAMMPS 8 February 2023) software platform [40], with post-simulation visualization of dynamic processes implemented in VMD.

**Figure 2:**
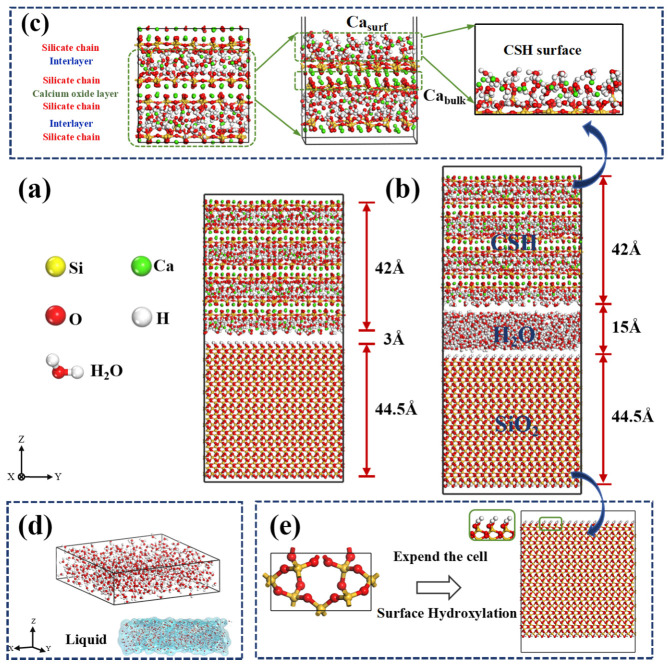
Model construction process: (**a**) the CSH/SiO_2_ interface model, (**b**) the CSH/H_2_O/SiO_2_ interface model, (**c**) the CSH matrix, (**d**) H_2_O, and (**e**) SiO_2_.

Calcium silicate hydrate (CSH), accounting for approximately 60% of the hydration products in cement matrices, is typically employed as a representative model for cement matrices in molecular dynamics simulations. Tobermorite and CSH gel molecular models share an analogous layered calcium silicate framework. By adjusting the degree of silicate chain polymerization and Ca/Si ratio, the structural characteristics of actual cement hydration products can be effectively simulated [41]. In this study, the initial CSH model was constructed based on the 11 Å tobermorite crystal structure, following the modeling protocols proposed by Pellenq et al. [42] and Hou et al. [43]. The Ca/Si ratio was adjusted to approximately 1.69 via random removal of bridging silicate tetrahedra. Subsequently, the Grand Canonical Monte Carlo (GCMC) method was utilized to intercalate water molecules into the interlayers of the dry and disordered CSH structure until the system density reached a saturated state. Then, the unit cell was expanded in the X, Y, and Z directions to construct a CSH supercell. The finalized CSH gel supercell has a chemical formula of (CaO)_1.69_(SiO_2_)(H_2_O)_1.8_, with dimensions of a = 44 Å, b = 43.8 Å, and c = 42 Å, and all interaxial angles equal to 90°. The supercell was cleaved along the [0, 0, 1] direction, perpendicular to the calcium-silicate layers, to expose the CSH substrate surface for subsequent interface modeling. The Ca atoms in the CSH surface model are classified into two types based on different chemical environments: active calcium atoms exposed on the surface (denoted as Ca_surf_) and bulk calcium atoms within the CSH gel (denoted as Ca_bulk_), as depicted in Figure 2c.

The rock surface was simulated using α-SiO_2_ crystals [8]. The α-SiO_2_ crystal was first subjected to unit cell expansion, followed by cleavage along the [0, 0, 1] plane to obtain an active surface with X and Y dimensions matching those of the CSH model. Hydrogen atoms were then grafted onto the oxygen atoms exposed on the surface layer to construct a hydrated surface containing silanol groups (Si-OH) [44,45], as depicted in Figure 2e. The SPC water model was adopted, as it balances high accuracy and computational efficiency in molecular simulations of cement-based materials [46], as shown in Figure 2d. After the pretreatment of each component, the CSH surface and the SiO_2_ surface were placed parallel to the *Z*-axis with an initial separation distance of 3 Å to construct the CSH/SiO_2_ direct-contact model (hereafter referred to as the CSH/SiO_2_ interface model), as depicted in Figure 2a. A vacuum gap of approximately 15 Å was introduced between the CSH and SiO_2_ surfaces, followed by the insertion of a free water layer to construct the CSH/H_2_O/SiO_2_ water-containing interface model (hereafter referred to as the CSH/H_2_O/SiO_2_ interface model), as depicted in Figure 2b.

### 2.2. Force Field and Simulation Details

**Figure 3:**
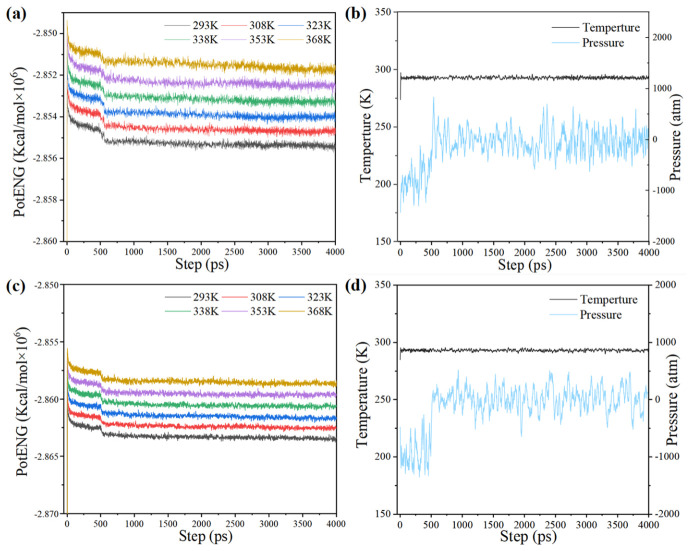
Trend of system properties during the equilibrium process: (**a**) Temperature, pressure, and (**b**) potential energy for the CSH/SiO_2_ interface model and (**c**) Temperature, pressure, and (**d**) potential energy for the CSH/H_2_O/SiO_2_ interface model.

The ClayFF force field has been widely utilized in the simulations of cement hydration products, as it enables accurate characterization of the structural properties, hydration behavior, and interfacial dynamics of silicate minerals. Thus, the ClayFF force field was selected as the computational force field in this study [35,43,47]. Temperature control was achieved via the Nose-Hoover thermostat, with a time step of 1 fs. To eliminate boundary effects, all simulations were conducted under periodic boundary conditions. Each interface model underwent an identical relaxation procedure at six target temperatures: 293 K, 308 K, 323 K, 338 K, 353 K, and 368 K. Initially, the energy minimization was performed using the conjugate gradient method to eliminate atomic overlaps in the initial configurations. This was followed by a 0.5 ns relaxation in the NVT ensemble to stabilize the system temperature. Finally, a 3.5 ns relaxation was conducted in the NPT ensemble with the Z-direction pressure set at 1 atm to bring the system to its final equilibrium state. Equilibration of the system was verified by monitoring the time-dependent fluctuations of energy, temperature, and pressure at 293 K, as shown in Figure 3. Based on the equilibrated models, a 1 ns production run was performed, with atomic coordinates and energy data recorded every 0.1 ps for subsequent structural and dynamic analysis.

### 2.3. Model Validation

**Figure 4:**
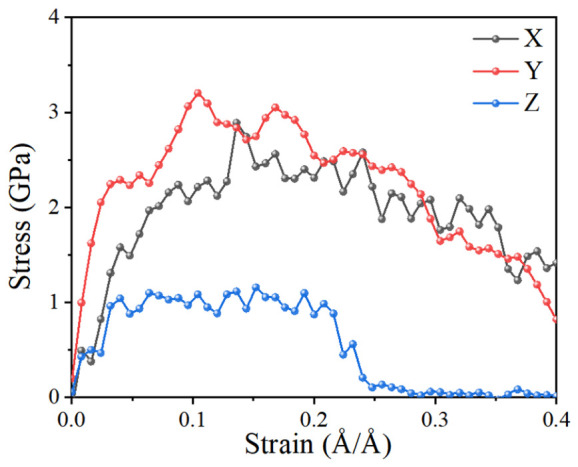
Stress–strain curves for the CSH.

To validate the reliability of the constructed CSH molecular model, the uniaxial tensile simulations were performed along the three principal directions of the model. The entire tensile simulation utilized the NPT ensemble with a time step of 0.1 fs, and the equations of motion were integrated using the Verlet algorithm. Before the uniaxial tensile tests, the model underwent thermodynamic relaxation: after energy minimization, it was relaxed for 300 ps at 300 K under zero pressure in all directions to eliminate residual internal stresses. Once the system reached equilibrium, uniaxial tension was applied sequentially along the three directions of the CSH model at a strain rate of 0.0008 ps^−1^. To account for the influence of Poisson’s ratio on the system stress during stretching, the pressures in the directions lateral to the loading axis were set to 0 atm. The resulting stress–strain curves are shown in Figure 4.

The mechanical behavior of CSH exhibits significant anisotropy, with the tensile strength following the order fy > fx > fz. The tensile strength along the *Z*-axis is 1.12 GPa, which is consistent with the range of 0.83–1.3 GPa reported in previous studies, as listed in Table 1. Furthermore, the stress–strain curve characterizes a brittle fracture, further validating the reliability of the model [33]. The CSH model exhibits higher mechanical strength in the X and Y directions, with tensile strengths of 2.4 GPa and 3.2 GPa, respectively. The calculated Young’s moduli in the X and Y directions are 39.5 GPa and 55.4 GPa, respectively, aligning well with the 50 GPa measured in nanoindentation experiments [48]. The tensile strength, Young’s modulus, and fracture evolution trends of the constructed CSH model are in good agreement with existing simulation and experimental results, validating the accuracy of the molecular model.

## 3. Simulation of Mechanical Properties

### 3.1. Simulation Procedure

In this section, uniaxial tensile simulations were performed along the *Z*-axis on the equilibrated interface models to investigate the effects of different temperature environments on the interfacial mechanical properties. Following the methodology reported in reference [50], uniform uniaxial tension was applied along the *Z*-axis of the models under the NVT ensemble, with a strain rate of 0.001 ps^−1^. The NVT ensemble was selected to suppress lateral deformation of the system and mitigate numerical instabilities arising from abrupt fluctuations in simulation box dimensions, thereby ensuring the convergence of stress calculations. This approach has been well validated in existing literature for studying interfacial behavior under uniaxial tension [51,52]. For each temperature condition, three uniaxial tensile tests were performed using different random seeds for initial velocities to ensure statistical reliability. The mechanical parameters reported below are the average values of these three parallel simulations.

### 3.2. Stress–Strain Curves

**Figure 5:**
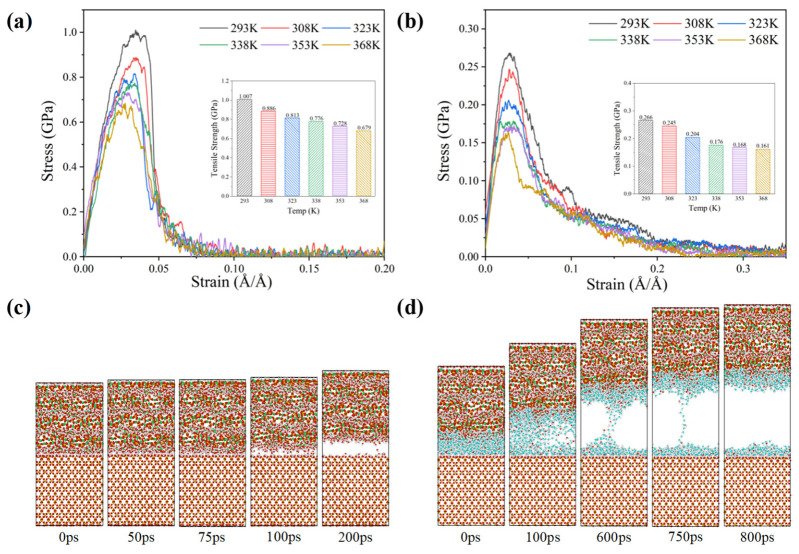
Tensile stress–strain curves of the composite structures under different temperatures: (**a**) the CSH/SiO_2_ interface and (**b**) the CSH/H_2_O/SiO_2_ interface; Atomic snapshots for the fracture process of the interface: (**c**) the CSH/SiO_2_ interface and (**d**) the CSH/H_2_O/SiO_2_ interface.

The molecular dynamics simulation results of the uniaxial tensile tests for the two composite models at varying temperatures are shown in Figure 5. The stress–strain curves of both models can be divided into three primary stages. In the early stage of tension, the composite structure undergoes minor deformation, accompanied by a rapid rise in internal stress. Subsequently, as the strain increases, the composite enters the yield stage, where the interfacial adhesion is maintained by multiple slender chain bridges. Finally, the composite reaches the damage stage, during which internal stress declines sharply with further increases in strain. A key distinction between the stress–strain curves of the two models lies in their post-peak behavior: the CSH/SiO_2_ interface model exhibits a relatively steep stress drop, whereas the CSH/H_2_O/SiO_2_ interface model displays a gradual downward trend in stress. The atomic snapshots in Figure 5c,d visually illustrate the differences in fracture behavior between the two interface models. In the CSH/SiO_2_ interface model, rapid interfacial delamination occurs within 200 ps, with damage primarily localized at the interface. This simulation observation is consistent with the experimental phenomenon reported by Djouani [15], confirming that the interface is the weak zone of the system. In contrast, the fracture process of the CSH/H_2_O/SiO_2_ interface model is significantly prolonged. During the 600–750 ps stage, the formation of nanoscale water bridges between the solid surfaces and subsequent cavitation can be observed.

The stress–strain curves of the CSH/SiO_2_ interface model are presented in Figure 5a. The interfacial tensile strength decreases with increasing temperature, dropping from 1.007 GPa at 293 K to 0.679 GPa at 368 K, representing a reduction of 32.57%. Furthermore, the strain corresponding to the stress peak shifts toward lower values. These observations indicate a significant thermal degradation of the interfacial bonding performance: high temperatures weaken the chemical bonding and van der Waals interactions connecting the CSH and SiO_2_, thereby rendering the interface more susceptible to fracture. With the introduction of a water layer, the mechanical strength of the interface is drastically reduced, while the temperature-induced weakening effect persists and exhibits a synergistic effect with the water layer. At 293 K, the introduction of the water layer causes the interfacial tensile strength to drop from 1.007 GPa to 0.266 GPa, a reduction of 73.58%. At 368 K, the strength of the water-containing interface falls to as low as 0.161 GPa, which is 76.29% lower than that of the direct-contact interface at the same temperature. These uniaxial tensile simulations can be interpreted as a macroscopic manifestation of microscopic mechanisms, where both high temperature and the intervention of water layers exert a deleterious impact on interfacial bonding.

## 4. Results and Discussion

### 4.1. Effect of Temperature on the Bonding Performance of the CSH/SiO_2_ Interface

#### 4.1.1. Interfacial Interaction Energy

**Figure 6:**
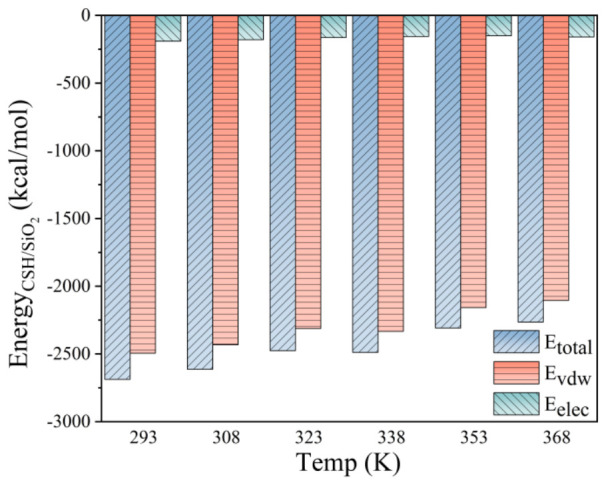
The interaction energy of CSH and SiO_2_.

The interaction energy is defined as the difference between the total energy of the system and the sum of the energies of its individual isolated components. A negative interaction energy indicates mutual attraction between the two components, and the magnitude of its absolute value directly correlates with the thermal stability of the interfacial bonding. Figure 6 illustrates the interaction energy between CSH and SiO_2_ at varying temperatures, providing an energy-based explanation and validation for the interfacial adhesion weakening effect under high-temperature conditions. The interfacial interaction energy reaches a maximum of −2687.05 kcal/mol at 293 K, then decreases with increasing temperature to −2263.27 kcal/mol at 368 K, representing a reduction of 15.78%. This trend is consistent with the variation in mechanical properties, suggesting that the interfacial instability caused by high temperature is the fundamental driving force for the degradation of mechanical properties. The total interaction energy (*E_total_*) encompasses electrostatic interactions (*E_elec_*) and van der Waals interactions (*E_vdw_*) [53]. At the CSH/SiO_2_ interface, vdW forces dominate, contributing over 90% of the total binding energy. According to the calculation formula for van der Waals interaction energy, the continuous decrease in *E_vdw_* implies that high temperatures induce an increase in atomic distances and interfacial expansion. Such atomic-scale loosening leads to stress concentration during loading, which in turn accelerates the initiation and propagation of cracks at the weak interface. In conclusion, the high temperatures consistently weaken the adhesive bonding performance of the CSH/SiO_2_ interface. The calculation formula for van der Waals forces is as follows:

(1) $$E_{vdw}=4\varepsilon \left[{\left(\frac{\sigma }{r_{ij}}\right)}^{12}-{\left(\frac{\sigma }{r_{ij}}\right)}^{6}\right],$$

where *ε* is the depth of the potential well, *σ* is the distance at which the inter-particle potential is zero, and *r_ij_* is the distance between atoms *i* and *j*.

#### 4.1.2. Atomic Density Distribution

**Figure 7:**
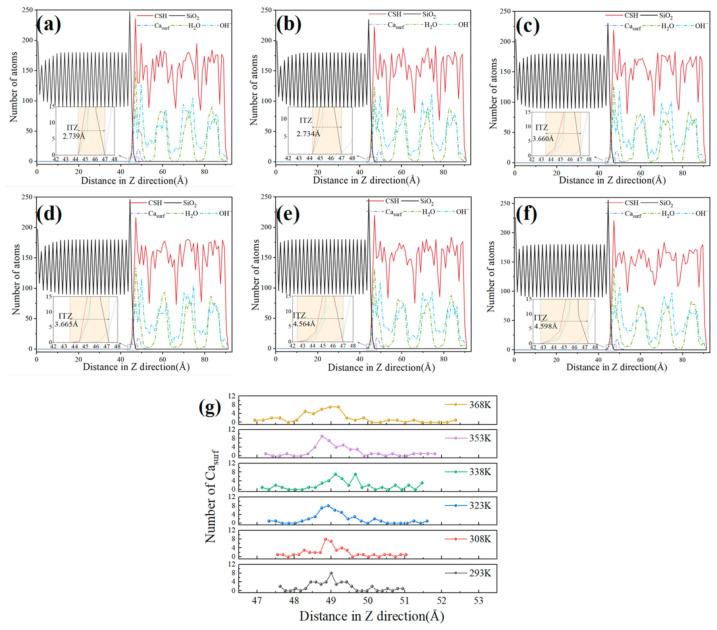
Atomic density distribution at the CSH/SiO_2_ interface: (**a**) 293 K, (**b**) 308 K, (**c**) 323 K, (**d**) 338 K, (**e**) 353 K, and (**f**) 368 K. (**g**) The density distribution of Ca_surf_ along the *z*-axis at different temperatures.

The atomic density distribution of each component along the *Z*-axis is presented in Figure 7. The SiO_2_ region on the left side of the density distribution curve exhibits periodic oscillations, consistent with its crystalline structural characteristics, while the CSH region on the right side shows a fluctuating amorphous distribution [54]. An interpenetrating zone exists between the CSH and SiO_2_ interfaces, which is defined as the interfacial transition zone (ITZ). A comparison of ITZ regions across different temperatures shows that the ITZ width expands with increasing temperature, rising from 2.739 Å at 293 K to 4.598 Å at 368 K. This phenomenon arises from the enhanced thermal motion of atoms at high temperatures, which increases atomic diffusion distances and promotes mutual penetration between CSH and SiO_2_ atoms, thereby broadening the ITZ. Notably, while atomic interpenetration would intuitively increase interfacial contact area to strengthen bonding, the interfacial interaction energy decreases with rising temperature. This seemingly contradictory observation will be elucidated in subsequent radial distribution function (RDF) and mean square displacement (MSD) analyses, integrating insights into bonding states and dynamic stability. The strong ionic bonds formed between the Ca_surf_ of CSH and the surface oxygen atoms of SiO_2_ constitute the core mechanism of interfacial bonding. Accordingly, this study further analyzed the density distribution of Ca_surf_ on the CSH surface at various temperatures. The simulation results indicate that when the temperature rises to 368 K, the peak of the calcium ions distribution shows a significant decrease in intensity and broadening of the peak shape. Ca_surf_ migrates not only toward the SiO_2_ surface but also into the interior of CSH. The intensified atomic positional fluctuations induced by thermal motion cause Ca_surf_ to detach from initial adsorption sites and lead to a more diffuse spatial distribution in the interfacial region, thereby reducing the probability of forming stable ionic bonds with specific oxygen atoms on the SiO_2_ surface.

#### 4.1.3. Volume Expansion

**Figure 8:**
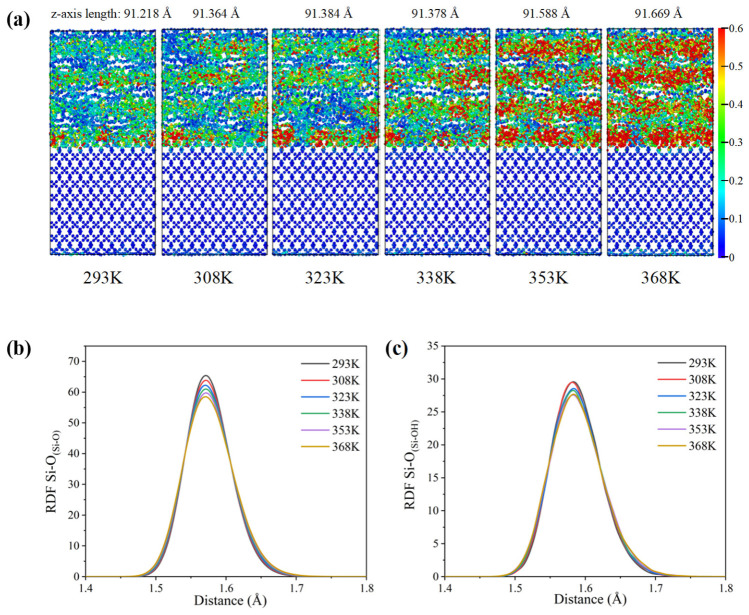
(**a**) Atomic Von Mises strain distribution at different temperatures, and the RDF curves of (**b**) Si–O_(Si-O)_ bonds and (**c**) Si–O_(Si-OH)_ bonds.

The Z-direction dimension of the simulation box gradually increases with rising temperature, and its expansion mechanism can be attributed to two key aspects. First, the interlayer regions undergo expansion as temperature increases, driving an overall increase in system volume. Figure 8a presents the cloud maps of atomic Mises strain distribution for the system at different temperatures. As the temperature rises, the distribution range of high-strain atoms within the CSH interlayer region expands significantly. The CSH gel interlayer is primarily filled with water molecules, and the number of interlayer water molecules remains nearly constant during heating (Figure 7), indicating that the volume expansion of this region is not caused by component migration, but rather by the enhanced kinetic energy of water molecules at high temperatures. This increases the average intermolecular distance between water molecules, which in turn pushes adjacent silicate layers apart, expanding the interlayer spacing and ultimately leading to volume expansion of both the interlayer region and the entire simulation system. This aligns closely with the mechanism of system volume expansion driven by thermal motion of interlayer water molecules under the constraint of the silicate skeleton, as proposed by Sun et al. [55]. Concurrently, the interfacial region also exhibits a trend of evolving from a low-strain, structurally dense state toward a high-strain, structurally relaxed state. This temperature-induced structural dilation at the nanoscale is consistent with previous studies on thermal volume expansion in both cement hydrates [56,57] and mineral phases [58].

The bond lengths of the silicate exert a critical influence on the volume of the CSH gel. Figure 8b,c present the radial distribution function (RDF) curves for the Si-O_(Si-O)_ and Si-O_(Si-OH)_ bonds within the CSH silicate chains. The bond lengths are determined by analyzing the position of the first coordination peak in the corresponding RDFs. Both bond lengths remain stable across the temperature range of 293 K to 368 K, maintaining values of 1.575 Å and 1.585 Å, respectively. However, the shoulder peaks of the RDFs for both bond types exhibit a gradual broadening of their distribution width with increasing temperature: the shoulder region of Si-O_(Si-O)_ bonds expands from 1.745 Å to 1.795 Å, while that of Si-O_(Si-OH)_ bonds widens from 1.765 Å to 1.795 Å. This observation indicates that some Si-O_(Si-O)_ and Si-O_(Si-OH)_ bonds undergo slight elongation at high temperatures [59], driving the three-dimensional stretching of silicate chains. Integrating the Mises strain contour maps with the RDF analysis of silicate chain bond lengths reveals that the entire composite structure undergoes gradual structural relaxation as temperature rises. The atoms become more dynamic and unstable, providing robust support for the conclusion of interfacial instability.

#### 4.1.4. Local Bonding Characteristics

The radial distribution function (RDF) is a pivotal analytical technique in molecular dynamics simulations for the quantitative characterization of the local atomic structure of a system. It can reveal the bond lengths and coordination numbers of ionic and hydrogen bonds (H-bonds) at the interface, thereby enabling a quantitative assessment of the strength and stability of interfacial bonding. A larger peak in the RDF curve indicates a higher interaction intensity between ion pairs, implying a greater probability of mutual attraction. Furthermore, the peak position is closely correlated with the chemical bond length: a smaller peak position value denotes a shorter bond length and a more stable configuration [60]. To conduct an in-depth microscopic analysis of the effect of temperature on the bonding of the CSH/SiO_2_ system, particularly those at the interface, this section calculates the RDFs for key atom pairs within the composite system.

**Figure 9:**
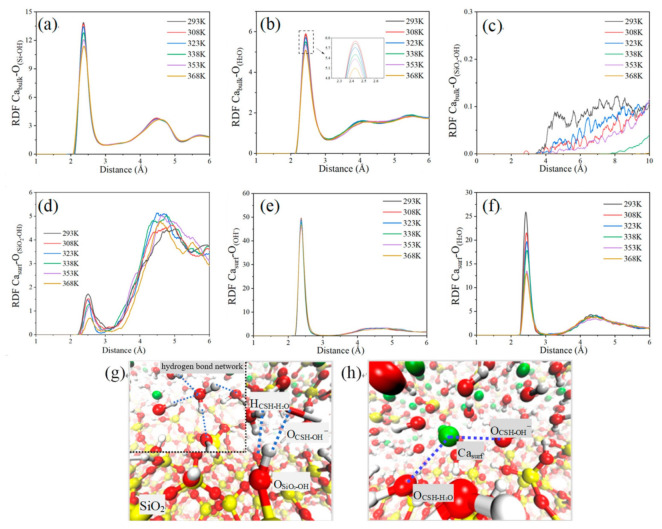
The RDF curves of (**a**) Ca_bulk_-O_(Si-OH)_, (**b**) Ca_bulk_-O_(H2O)_, (**c**) Ca_bulk_-O_(SiO2-OH)_, (**d**) Ca_surf_-O_(SiO2-OH)_, (**e**) Ca_surf_-O_(OH^−^)_, and (**f**) Ca_surf_-O_(H2O)_ under temperatures ranging from 293 K to 368 K; The snapshots of (**g**) CSH/SiO_2_ bonding interface, (**h**) Ca_surf_-O_CSH_ bonds.

In the interior of CSH, Ca atoms form coordination polyhedra with non-bridging oxygen atoms on the silicate chains, linking discrete silica tetrahedra into an integrated structure and thus maintaining the structural stability of the CSH gel [61]. Figure 9a,b present the RDFs between Ca_bulk_ and both the non-bridging oxygen atoms and water molecules within the CSH internal region. The results show that the bond lengths of Ca_bulk_-O_(Si-OH)_ and Ca_bulk_-O_(H2O)_ remain stable across the temperature range of 293 K to 368 K, at approximately 2.335 Å and 2.425 Å, respectively. This is consistent with the reported common distance of 2.35 Å for Ca-O ion pairs by Hosseini et al. [62]. However, the RDF peak intensities of Ca_bulk_-O_(Si-OH)_ and Ca_bulk_-O_(H2O)_ bonds decrease with increasing temperature, indicating that high temperatures reduce the connectivity between silicate tetrahedra in the CSH gel and compromise the intrinsic structural stability of the CSH.

Figure 9c displays the RDF values between Ca_bulk_ and the hydroxyl oxygen atoms on the SiO_2_ surface. No distinct characteristic coordination peaks were observed within a statistical radius of 10 Å, confirming that Ca_bulk_ atoms do not participate in interfacial bonding. Figure 9d illustrates the RDF curves between the Ca_surf_ of CSH and the hydroxyl oxygen atoms on the SiO_2_ surface. The bond length of Ca_surf_-O_(SiO2-OH)_ is 2.505 Å at 293 K; as the temperature increases to 368 K, the bond length gradually increases, reaching 2.555 Å. Longer bond length corresponds to lower bond strength, indicating that high temperature exerts a degrading effect on the Ca_surf_-O_(SiO2-OH)_ bond strength. Concurrently, the peak intensity of the RDF curve decreases from 1.72 at 293 K to 0.69 at 368 K, suggesting that high-temperature environments inhibit the binding between surface Ca ions and hydroxyl oxygen atoms on the SiO_2_ surface. The position and intensity of the first peak of the Ca_surf_-O_(SiO2-OH)_ bond are both lower at high temperatures than at low temperatures, confirming that high temperatures weaken the bonding between SiO_2_ surface hydroxyl groups and Ca_surf_. Similarly, as depicted in Figure 9e,f, the RDF peak intensities for Ca_surf_-O_(OH^−^)_ and Ca_surf_-O_(H2O)_ also exhibit a pronounced decline with increasing temperature, further demonstrating that thermal effects universally weaken the coordination capacity of surface calcium ions. Figure 9g,h provide bonding snapshots of Ca_surf_ with hydroxyl oxygen atoms on the SiO_2_ surface, as well as with free hydroxyl groups and water molecules.

**Figure 10:**
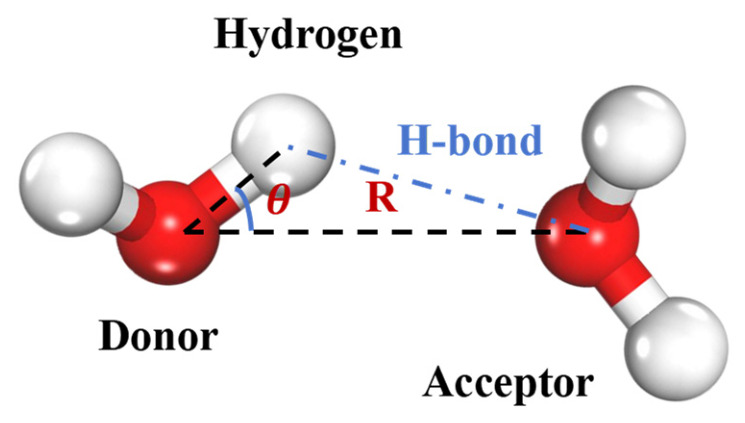
Mechanism of H-bond formation.

Beyond the Ca-O ionic bonds mediating interfacial adhesion, the hydrogen bond network formed between CSH and SiO_2_ also modulates the interfacial bonding performance. The formation of an H-bond must simultaneously satisfy two criteria: the distance between a donor oxygen atom and an acceptor oxygen atom must be less than 2.45 Å, and the corresponding α angle between the donor, hydrogen, and acceptor must be less than 30°, as illustrated in Figure 10 [63].

**Figure 11:**
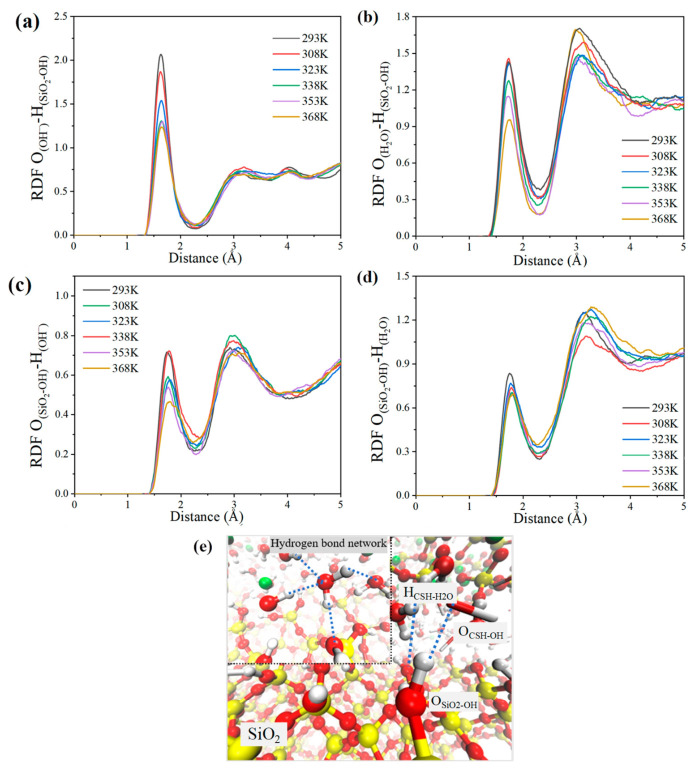
The RDF curves of (**a**) O_(OH^−^)_-H_(SiO2-OH)_, (**b**) O_(H2O)_-H_(SiO2-OH)_, (**c**) O_(SiO2-OH)_-H_(OH^−^)_, and (**d**) O_(SiO2-OH)_-H_(H2O)_; (**e**) The snapshot of H-bonds in the interfacial region.

Figure 11a–d present the RDFs of H-bonds formed between the CSH matrix and the SiO_2_ surface hydroxyl groups acting as O-acceptors and O-donors across different temperature conditions. The first coordination peaks of O_(OH^−^__)_-H_(SiO2-OH)_, O_(H2O)_-H_(SiO2-OH)_, O_(SiO2-OH)_-H_(OH^−^__)_, and O_(SiO2-OH)_-H_(H2O)_ all fall within the range of 1.6 Å to 1.8 Å. These distances are significantly smaller than the geometric threshold of 2.45 Å for H-bond formation, confirming the presence of a hydrogen bond network between the SiO_2_ and CSH components, as shown in Figure 11e. When the SiO_2_ surface hydroxyl group acts as an oxygen donor, the first peak position of the RDF formed with the CSH surface hydroxyl oxygen occurs at 1.625 Å, which appears earlier than the peak position of 1.735 Å in the O_(H2O)_-H_(SiO2-OH)_ curve. The bond length of O_(OH^−^__)_-H_(SiO2-OH)_ is shorter than that of O_(H2O)_-H_(SiO2-OH)_, indicating that H_(SiO2-OH)_ exhibits a stronger preference for binding with O_(OH^−^__)_ atoms rather than O_(H2O)_ atoms. In the range of 3.0–5.0 Å, all RDFs exhibit distinct second coordination peaks. This medium-range disorder suggests a strong spatial correlation between the CSH matrix and SiO_2_ in the interfacial region [64].

As the temperature increases from 293 K to 368 K, the first peak positions of all H-bonds exhibit a slight rightward shift. Taking the RDF curve of O_(SiO2-OH)_-H_(OH^−^__)_ as an example, its first peak shifts from 1.625 Å rightward to 1.655 Å, indicating that not only the strength of the Ca-O bond decreases, but the strength of the H-bonds also diminishes. This observation allows the inference that high temperatures enhance the thermal motion of atoms, causing them to deviate from their original equilibrium positions and thereby leading to longer average bond lengths [65]. Subsequently, analyzing the evolution of peak intensities reveals a monotonically decreasing trend in the first peak values of RDF curves for all component pairs as temperature rises. Coordination numbers can be quantified by the area under the first RDF peak. It is evident that the bond coordination numbers at high temperatures are consistently lower than those at low temperatures across all four plots. This indicates that high temperatures reduce both the quantity and stability of effective H-bonds. Thus, it can be concluded that under high-temperature conditions, the H-bonds formed between SiO_2_ surface hydroxyl groups and the CSH matrix undergo a reduction in both number and strength. The systematic degradation of interfacial H-bond strength explains the decline in macroscopic interfacial adhesion performance.

**Figure 12:**
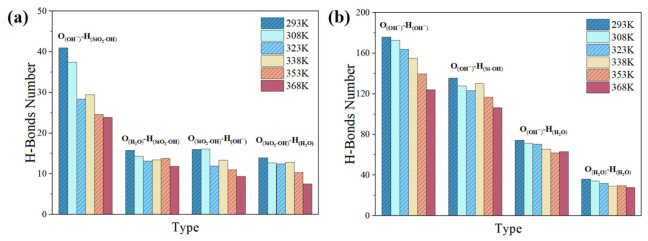
(**a**) The number of H-bonds between the CSH and SiO_2_. (**b**) The number of H-bonds within the CSH matrix.

Figure 12 displays the temperature-dependent variations in the number of distinct H-bond types within the CSH/SiO_2_ system, which complements and cross-validates the RDF findings. Specifically, Figure 12a reveals that within the CSH/SiO_2_ interfacial region, the quantity of O_(OH^−^__)_-H_(SiO2-OH)_ bonds is significantly higher than that of O_(H2O)_-H_(SiO2-OH)_ bonds across all temperatures. When the hydroxyl groups on the SiO_2_ surface act as donors, they preferentially bond with the hydroxyl oxygen atoms on the CSH surface, which is consistent with the conclusions from the previous RDF analysis. Figure 12b displays the number of H-bonds within the CSH matrix, totaling 419, which is far greater than the 80 bonds observed in the interfacial region. This distribution characteristic, where the bulk-phase bond count is significantly higher than that of the interface, reflects the interface as the mechanical weak link of the composite model from a chemical bonding perspective. This observation also provides a rational explanation for the phenomenon that fracture surfaces in uniaxial tensile simulations are localized to the interfacial region.

As the temperature increases from 293 K to 368 K, the abundance of all H-bond types decreases consistently. Notably, the O_(OH^−^__)_-H_(SiO2-OH)_ bonds, which represent the primary contributors to interfacial adhesion, exhibit the most dramatic decline, dropping from 40.97 to 23.92, representing a 41.6% reduction. High temperatures significantly amplify the amplitude of atomic thermal vibrations, inducing pronounced deviations in the spatial geometry of hydrogen bond donor–acceptor pairs. Consequently, a substantial number of atom pairs fail to simultaneously satisfy the dual geometric criteria for H-bond formation. A substantial decrease in the core interfacial H-bonds weakens the interfacial interaction energy, ultimately manifesting macroscopically as an overall decline in the interfacial tensile performance. In contrast to the hydroxyl H-bonds at the interface, the number of H-bonds involving water molecules fluctuates slightly with temperature. Water molecules possess structural features of small volume and high rotational freedom. Upon the cleavage of original H-bonds at high temperatures, water molecules can rapidly recombine with new H-bond acceptors via dynamic reorganization, thereby maintaining a statistically stable number of H-bonds.

#### 4.1.5. Dynamic Characteristics

Based on the statistics of RDFs and the number of H-bonds, this paper quantitatively reveals the static effect of temperature on the bonding structure at the CSH/SiO_2_ interface. However, interfacial bonding is not static, and its dynamic stability also plays a critical role in interfacial adhesion performance. In this section, mean square displacement (MSD) analysis and time correlation function (TCF) analysis are employed.

MSD is used to compute the mean value of the squared displacement of particles relative to their initial positions over a specified time interval. The diffusion coefficient is positively correlated with the slope of the linear region of the MSD curve. A steeper slope of the MSD curve corresponds to a higher diffusion coefficient, indicating more pronounced diffusion behavior of the particles and greater migration capacity at the microscopic scale.

(2) $$M(t)=\langle {\left|r_{i}(t)-r_{i}(0)\right|}^{2}\rangle$$

Here, *r_i_*(0) represents the initial position of the particle, *r_i_*(*t*) represents the position of the same particle at time *t*, and the angular brackets denote the ensemble average over all particles of the same type.

**Figure 13:**
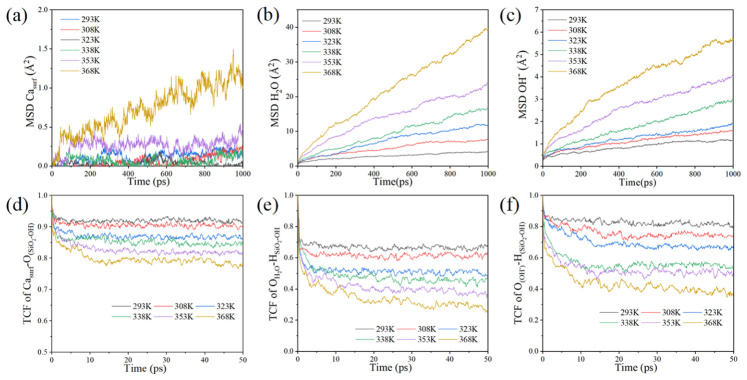
MSD of (**a**) Ca_surf_, (**b**) H_2_O, and (**c**) OH^−^; TCF of (**d**) Ca_surf_-O_(SiO2-OH)_, (**e**) O_(H2O)_-H_(SiO2-OH)_, and (**f**) O_(OH^−^)_-H_(SiO2-OH)_.

As shown in Figure 13a–c, when the temperature rises from 293 K to 368 K, the slopes of the MSD curves for all particle types rise significantly. This indicates that thermal excitation effectively overcomes the binding energy exerted by the CSH framework on atoms, thereby markedly enhancing the random thermal motion of the particles. Distinct disparities exist in the diffusion rates of different particles, with the migration capacity ordered from highest to lowest as follows: H_2_O > OH^−^ > Ca_surf_. Under the same temperature conditions, water molecules exhibit the fastest diffusion rate. Their small molecular mass, high rotational freedom, and the rapid dynamic equilibrium of the hydrogen bond network collectively enable them to maintain extremely high dynamic activity at the interface. This also results in the number and strength of H-bonds formed between water molecules and the hydroxyl groups on the SiO_2_ surface being lower than those of the hydroxyl groups themselves. The excessively high diffusion rate inherently renders these bonds unstable and overly dynamic.

At low temperatures, the MSD values of Ca_surf_ are relatively small, reflecting the formation of strong chemical bonds between Ca_surf_ and the CSH matrix. Consequently, over longer time scales, Ca_surf_ primarily undergoes in situ oscillation or rotational motion around its equilibrium positions. An increase in temperature enhances the mobility of Ca_surf_ through two mechanisms: first, the kinetic energy of Ca_surf_ itself increases; second, the rapid diffusion of interfacial water molecules induces continuous collisions and solvation disturbances. Under the synergistic effect of these two factors, Ca_surf_ is more prone to detaching from its original adsorption sites, leading to a substantial reduction in the probability of forming stable ionic bonds with oxygen atoms on the SiO_2_ surface and ultimately weakening the stability of interfacial bonding.

TCF was used to quantitatively describe the stability of a chemical bond. The normalized bond length autocorrelation function, C(t), is defined as:

(3) $$C(t)\;=\frac{<\delta b(t)\delta b(0)>}{<\delta b(0)\delta b(0)>}$$

where *δb*(*t*) = *b*(*t*) − <*b*>, and *b*(*t*) is a binary variable indicating whether the bond is intact (1) or broken (0) at time *t*. A higher TCF value means a more stable chemical bond.

Figure 13d–f illustrate the TCF curves of various chemical bonds at different temperatures. With increasing temperature, the TCF values of all bond types decrease significantly, indicating a gradual reduction in bond stability. Coupled with the higher atomic diffusion rates observed in the MSD at high temperatures, atoms are more prone to detaching from their bonded sites, thereby further undermining bond stability. Based on the TCF values across different temperatures, the stability order of the chemical bonds is determined as: Ca_surf_-O_(SiO2-OH)_ > O_(OH^−^__)_-H_(SiO2-OH)_ > O_(H2O)_-H_(SiO2-OH)_. This stability hierarchy corroborates prior atomistic simulations on cementitious and mineral components [35]. The Ca-O ionic bond demonstrates the highest stability, with its energy substantially exceeding that of H-bonds. Among the oxygen atoms involved in hydrogen bonding, the hydroxyl-derived O_(OH^−^__)_ forms more stable hydrogen bonds compared to the water molecule-derived O_(H2O)_.

### 4.2. Effect of the Water Layer on the Bonding Performance of the CSH/SiO_2_ Interface

#### 4.2.1. Interaction Energy

**Figure 14:**
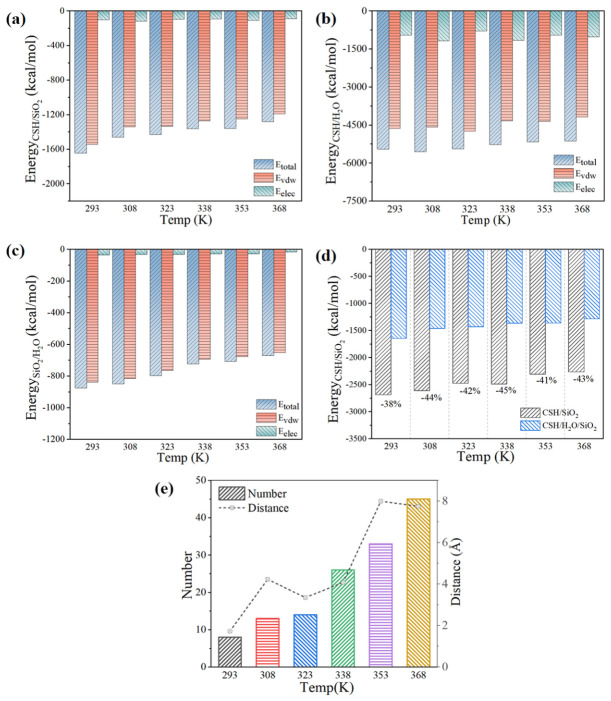
The interaction energy of (**a**) CSH/SiO_2_, (**b**) CSH/H_2_O, and (**c**) SiO_2_/H_2_O. (**d**) Comparison of CSH/SiO_2_ interfacial interaction energies for the two models. (**e**) The number of water molecules entering CSH and their penetration depth.

To investigate the differences in bonding performance at the CSH/SiO_2_ interface under varying temperatures and the presence of water layers, the interaction energies of components at the CSH/H_2_O/SiO_2_ interface were calculated, as presented in Figure 14a–c. A comparison of the interaction energies between CSH/SiO_2_, CSH/H_2_O, and SiO_2_/H_2_O reveals that the interaction energy between CSH and the water layer is significantly higher than that of CSH/SiO_2_ and SiO_2_/H_2_O. Taking the 293 K environment as an example, E_CSH/H2O_ reaches −5449 kcal/mol, which is substantially greater than E_CSH/SiO2_ = −1645 kcal/mol and E_SiO2/H2O_= −875 kcal/mol. This strong interaction between CSH and the aqueous solution constitutes the primary driving force for the degradation of interfacial bonding. When water molecules are attracted by CSH and accumulate at the interface, they not only act as a physical barrier but also block direct bonding between the two phases by preoccupying the active sites that originally belonged to the CSH and SiO_2_. Notably, the CSH/H_2_O interaction energy exhibits a relatively small decrease with increasing temperature, demonstrating stability. By correlating this observation with the water molecule distribution in Figure 14e, it can be inferred that high temperatures provide water molecules with sufficient kinetic energy to overcome local potential barriers, driving their deep intrusion into the CSH matrix. The increase in the number of intruding water molecules macroscopically compensates for the attenuation of individual bond energies, maintaining the strong adsorption state of CSH toward water molecules. This sustained occupation of CSH/SiO_2_ interfacial sites by water molecules ultimately leads to a significant loss of interfacial interaction energy. Figure 14d compares the interfacial interaction energies of the CSH/SiO_2_ interface in two models (with and without a water layer). The presence of water reduces the interfacial interaction energy at all temperatures by 38–45%, confirming the formation of an energy barrier effect by the water layer at the interface. In summary, the strong hydrophilicity of CSH drives the initial competitive adsorption of water molecules from the solution, and increasing temperature further facilitates the deep intrusion of water molecules, ultimately resulting in the loss of interfacial interaction energy.

#### 4.2.2. Local Bonding Characteristics

**Figure 15:**
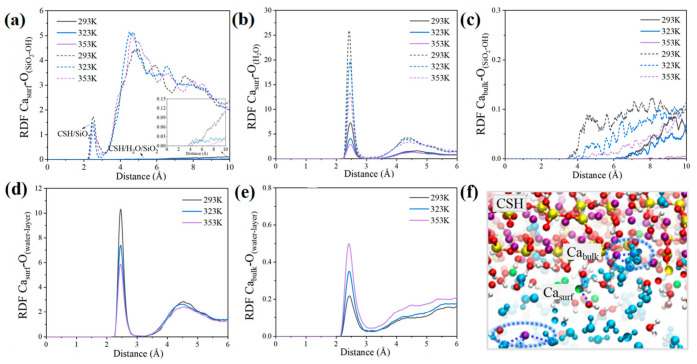
The RDF curves of (**a**) Ca_surf_-O_(SiO2-OH)_, (**b**) Ca_surf_-O_(H2O)_, (**c**) Ca_bulk_-O_(SiO2-OH)_, (**d**) Ca_surf_-O_(water-layer)_, and (**e**) Ca_bulk_-O_(water-layer)_ at different temperatures. (**f**) The snapshot shows the coordination environment of Casurf and Cabulk at the CSH interface.

The comparison of interaction energies has demonstrated that the water layer impairs the bonding performance at the CSH/SiO_2_ interface. To uncover the microscopic origin of this energy variation, the RDF of key atomic pairs at the interface was further computed in this section. Figure 15a presents the RDF of Ca_surf_-O_(SiO2-OH)_ under direct contact and water-containing conditions. For the CSH/SiO_2_ interface model, a strong, distinct first coordination peak is observed at 2.4 Å, indicative of the formation of robust Ca-O ionic bonds across the interface. Upon introduction of the water layer, the intensity of this coordination peak diminishes significantly, even nearly vanishing, directly confirming at the atomic scale that water molecules in solution effectively block direct bonding between Ca_surf_ and oxygen atoms on the SiO_2_ surface. Conversely, as shown in Figure 15c, no characteristic coordination peak is identified for Ca_bulk_-O_(SiO2-OH)_ within a 10 Å radius, confirming that Ca_bulk_ within the CSH matrix does not participate in interfacial bonding.

As shown in Figure 15b, under anhydrous conditions, Ca_surf_ and internal water molecules within CSH exhibit a clear coordination peak around 2.5 Å, corresponding to the stable local hydration structure in the direct contact model. Following the introduction of the interfacial water layer, the intensity of the RDF peak also decreases. When combined with the RDF peak of Ca_bulk_ and solution water molecules in Figure 15e, this reflects the global nature of the competitive effect exerted by solution water molecules. After infiltrating the interface, external water molecules not only compete with SiO_2_ for Ca sites on the CSH surface but also engage in competitive coordination with the original bound water within the CSH matrix [38]. The RDF curve of Ca_surf_-O_(water-layer)_ in Figure 15d shows a maximum peak at 2.45 Å, indicating that water molecules are attracted by the CSH matrix and form aggregates, with Ca_surf_-O_(water-layer)_ chemical bonds established between CSH and molecules. Consequently, some positions originally occupied by O_(SiO2-OH)_ are replaced by O_(H2O)_ from water molecules, leading to the substitution of partial Ca_surf_-O_(SiO2-OH)_ bonds with Ca_surf_-O_(water-layer)_ bonds and ultimately weakening the interaction between SiO_2_ and the CSH matrix. These competitive bonding states and local coordination environments surrounding both Ca_surf_ and Ca_bulk_ at the interface are intuitively visualized in the atomic snapshot in Figure 15f. It should be noted that the present simulations consider pure water under static conditions, without explicitly accounting for dissolved ions, external pressure, hydrodynamic flow, or geostress. In real underground environments, these factors may further influence interfacial bonding characteristics.

**Figure 16:**
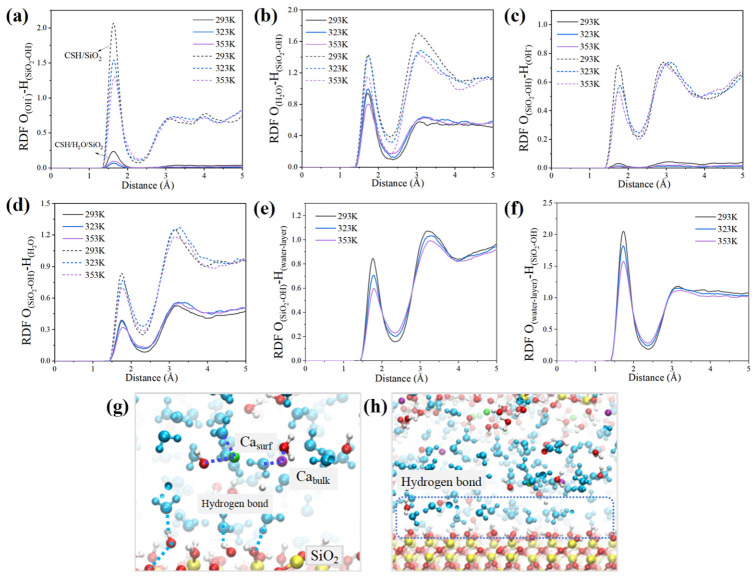
The RDF curves of (**a**) O_(OH^−^)_-H_(SiO2-OH)_, (**b**) O_(H2O)_-H_(SiO2-OH)_, (**c**) O_(SiO2-OH)_-H_(OH^−^)_, (**d**) O_(SiO2-OH)_-H_(H2O)_, (**e**) O_(SiO2-OH)_-H_(water-layer)_, and (**f**) O_(water-layer)_-H_(SiO2-OH)_; (**g**,**h**) The snapshots of H-bonds in the interfacial region.

With the introduction of the water layer, the RDF peaks corresponding to bonds such as O_(OH^−^)_-H_(SiO2-OH)_ and O_(H2O)_-H_(SiO2-OH)_ are notably lower than those in the direct contact state, as illustrated in Figure 16a–d. This confirms that the intrinsic hydrogen bond network at the interface is substantially weakened by the infiltration of water molecules. Conversely, the high RDF peaks between external water molecules and SiO_2_ surface hydroxyl groups, shown in Figure 16e,f, indicate that solution water molecules preferentially occupy the active sites on the SiO_2_ surface, leveraging their kinetic advantages. The competitive displacement of CSH/SiO_2_ H-bonds by the aforementioned solution water molecules forms a dynamic physical barrier at the interface. This barrier not only constructs an energy barrier from the energetic perspective but also structurally disrupts the direct bonding between CSH and SiO_2_, ultimately resulting in the systematic degradation of interfacial bonding performance under high-temperature, water-rich conditions. As illustrated in Figure 16g,h, a large number of active sites on the SiO_2_ surface are occupied by water molecules, further visually validating the described interfacial structural evolution.

**Figure 17:**
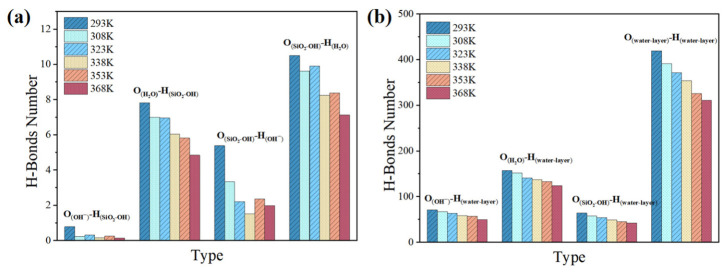
(**a**) The number of H-bonds between the CSH and SiO2, and (**b**) the number of H-bonds between the water layer and the substrates.

To quantitatively assess the hydrogen bonding between CSH, SiO_2_, and water molecules in solution, this section computes the H-bond counts at the CSH/SiO_2_ interface and between each substrate and the aqueous phase. The results are illustrated in Figure 17. The number of H-bonds formed between the solution water molecules and the CSH or SiO_2_ is far greater than the number of direct H-bonds at the CSH/SiO_2_ interface. Meanwhile, a comparison with the calculation results of the direct contact model in Section 4.1 reveals that the introduction of a water layer leads to a significant reduction in the number of direct interfacial H-bonds between CSH and SiO_2_. This explains the previously mentioned energy barrier mechanism from the perspective of interfacial coordination competition. Water molecules in the solution compete for and occupy active sites on the surfaces of CSH and SiO_2_, forming H-bonds such as O_(OH^−^)_-H_water-layer_ and O_(SiO2-OH)_-H_water-layer_. This competitive occupation inhibits the formation of H-bonds between SiO_2_ and CSH, ultimately impairing interfacial adhesion.

#### 4.2.3. Dynamic Characteristics

**Figure 18:**
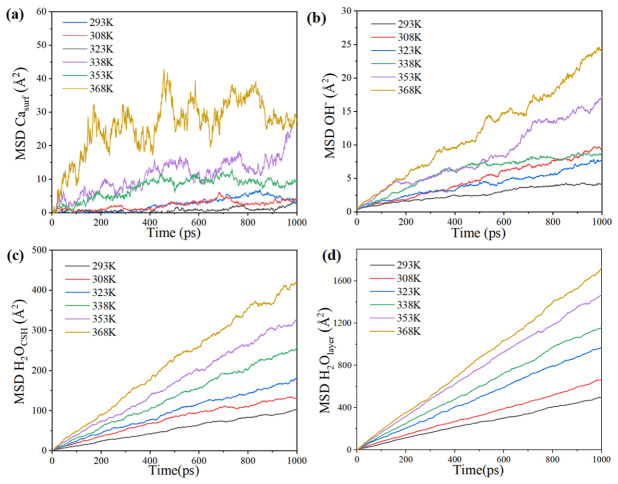
MSD of (**a**) Ca_surf_, (**b**) OH^−^, (**c**) H_2_O_CSH_, and (**d**) H2O_layer_.

In this section, the MSD values of Ca_surf_, H_2_O_CSH_, OH^−^, and water molecules in the external solution were calculated at different temperatures, as shown in Figure 18. Consistent with the CSH/SiO_2_ interface model, the diffusion rates of the target atoms increase with increasing temperature. It is noteworthy that the MSD values of Ca_surf_, H_2_O_CSH_, and OH^−^ are all higher than those in the CSH/SiO_2_ interface model. Taking 368 K as an example, their MSD values increased from 1 Å^2^, 40 Å^2^, and 5.5 Å^2^ to 30 Å^2^, 400 Å^2^, and 25 Å^2^, respectively, while the MSD of external water molecules reached as high as 1600 Å^2^. From these observations, it can be inferred that the introduction of the water layer accelerates the diffusion of all atoms/molecules involved. Due to their high kinetic activity, water molecules from the introduced layer can rapidly penetrate the voids at the CSH/SiO_2_ interface, competing for and occupying the active sites on both the CSH surface (e.g., Ca_surf_ and OH^−^) and the SiO_2_ surface. This competitive occupation thereby further undermines the stability of interfacial bonding.

## 5. Conclusions

In this paper, molecular dynamics simulations were conducted to systematically investigate the bonding performance and interfacial integrity of the CSH/SiO_2_ interface under the coupled influence of high temperatures and aqueous environments. This study elucidates the nanoscale degradation mechanisms governing the adhesion stability of cement-based materials, providing a theoretical foundation for the molecular design of resilient binder–substrate systems.

(1) A distinct thermal degradation effect has been revealed on the tensile strength of the CSH/SiO_2_ interface, where the tensile strength decreases with increasing temperature. The introduction of an interfacial water layer further degrades the interfacial tensile strength, which decreases by 73.58% at 293 K compared with the direct-contact interface. The presence of the interfacial water layer alters the interaction between CSH and SiO_2_, transforming it from direct interfacial interaction into indirect interaction mediated by interfacial water molecules.
(2) High temperature weakens the interface through multiple mechanisms. The interfacial interaction energy between CSH and SiO_2_ exhibits a monotonic decrease with increasing temperature. At 368 K, the E_CSH/SiO2_ value is 15.78% lower than that at 293 K, a trend consistent with the findings from uniaxial tensile simulations. High temperatures intensify atomic thermal vibrations, leading to a significant expansion of the interfacial transition zone thickness from 2.74 Å to 4.60 Å and a reduction in interfacial structural density.
(3) The interfacial core bonding is significantly weakened by high temperatures. High temperature reduces the number and stability of coordination bonds between Ca_(CSH)_ and O_(SiO2)_. For all types of H-bonds, their peak positions shift rightward, and both peak intensities and coordination numbers exhibit a decreasing trend. Accordingly, in grouting engineering for high-temperature fractured rock masses, the degradation of grout–rock interface performance of grouting materials under high-temperature conditions should be fully considered during material selection and formulation design. Priority should be given to grouting materials with excellent high-temperature resistance.
(4) The presence of an interfacial water layer is a critical factor leading to the degradation of the bonding behavior at the CSH/SiO_2_ interface. Compared with the direct-contact interface, the interaction energy of the CSH/SiO_2_ interface in an aqueous environment is reduced by 38% at 293 K. The strong hydrophilicity of CSH promotes the adsorption of water molecules from the solution onto its surface and even into the interior of CSH. These water molecules occupy interfacial active sites and hinder direct bonding between CSH and SiO_2_. High temperature facilitates the penetration depth and quantity of water molecules, further weakening the direct bonding at the CSH/SiO_2_ interface. Therefore, in grouting engineering, various measures should be adopted to improve the filling degree of grout in rock fractures, such as increasing grouting pressure, so as to reduce the water content at the grout–rock interface and enhance its interfacial performance.
(5) High temperatures provide kinetic energy to atoms via thermal excitation, enabling them to overcome the confinement of the CSH framework and significantly amplifying the random thermal motion of particles. This substantially reduces the probability of forming stable ionic bonds with oxygen atoms on the SiO_2_ surface. The intercalation of water layers further accelerates the thermal diffusion of interfacial particles, as evidenced by a marked increase in the MSD values of key atoms (e.g., Ca_surf_). The exceptionally high kinetic activity of water molecules in the solution allows them to infiltrate the CSH/SiO_2_ interfacial voids more rapidly, diminishing the interfacial bonding stability from a dynamic perspective. The synergistic effect of high temperature and water, operating through both static bonding degradation and dynamic diffusion enhancement, collectively drives the systematic deterioration of the grout–rock interface.

## Figures and Tables

**Table 1:** Mechanical properties of CSH under uniaxial tensile loading in the Z-direction.

MD Simulations	Water/Si	Ca/Si	Strain Rate (ps^−1^)	Tensile Strength (GPa)	Young’s Modulus (GPa)
Hou’s results [33]	1.5	1.6	0.0008	0.87	25.9
Zhang’s results [35]	1.49	1.67	0.0008	0.93	/
Zhou’s results [49]	1.70	1.65	0.008	1.38	26.23
This work’s results	1.8	1.69	0.0008	1.12	24.89

## Data Availability

The original contributions presented in this study are included in the article. Further inquiries can be directed to the corresponding author.
